# Analysis of disponible neuropsychological tests for early diagnosis of Alzheimer's disease: A systematic review

**DOI:** 10.1002/alz70857_095930

**Published:** 2025-12-23

**Authors:** Joabe Vasconcelos Silva, Gabrielle Guindani Maia, Luís Humberto Romeiro Tenório, Henrique Silva Lovera

**Affiliations:** ^1^ Universidade Federal de Ciências da Saúde, Porto Alegre, Rio Grande do Sul, Brazil; ^2^ Fundação Universidade Federal de Ciências da Saúde de Porto Alegre, Porto Alegre, Rio Grande do Sul, Brazil

## Abstract

**Background:**

With the global change of demographics towards an increase of the elderly population, diseases related to some form of dementia have become more common in the 21st century. Leading the hall of dementia problems is Alzheimer's Disease (AD), responsible for more than 50% of the reported cases. It is also known that early diagnosis helps to lead to a better prognosis. So, this review aims to evaluate the role of disponible neuropsychological tests (NT) on early diagnosis of AD, using the available literature, in order to determine the importance of its use.

**Method:**

An active search was performed in the PubMed database, using the keywords “Alzheimer's disease”, “early diagnosis”, “neuropsychological” and their equivalents. All articles published from 1998 to 2024 in any language were selected. The title and abstract were analyzed, duplicate results were eliminated and all studies that had the evaluation of the use of NT on early diagnosis of AD as their objective were included. Studies that analyzed only other types of dementia or non‐psychological tests were excluded.

**Result:**

The search yielded 77 articles, with 9 meeting the inclusion criteria. Findings reveal that various NTs show promise for early AD diagnosis, with tools like the Brain Health Assessment‐Cognitive Score and the DMS48 visual recognition memory test effectively identifying early cognitive impairment. The DMS48, in particular, highlighted that mild cognitive impairment patients with visual memory deficits face a faster cognitive decline, indicating a higher AD progression risk. The visual oddity detection task also proved effective, and combining the Mini‐Mental State Examination with other NTs, such as the Addenbrooke's Cognitive Examination, in screening was especially useful. Additionally, verbal fluency tests have shown several impairments of the early phase of AD and culturally adaptable tools like the Cross‐Cultural Neuropsychological Test Battery offered reliable assessment across diverse populations.

**Conclusion:**

The literature on the use of NT on early diagnosis of AD lacks data, no randomized clinical trial or large analytical observational study was found. However, among the information analyzed, the results suggest that combining the neuropsychological measures alongside biomarkers and informant‐based assessments enhances early AD detection and diagnostic accuracy.

